# Long-term effects of adolescent risperidone treatment on the mouse cortex

**DOI:** 10.1038/s41386-026-02483-2

**Published:** 2026-07-08

**Authors:** Abneil D. Alicea-Pauneto, Hyowon Choi, Wenyu Zhang, Xinjian Li, Laurence. A. Busque, Mingxuan Li, Andrew Wu, Evan Zhang, Adam D. Marc, Jonathan Preall, Kuan Hong Wang, Robert E. Featherstone, Steven J. Siegel, Chang-Gyu Hahn, Karin E. Borgmann-Winter

**Affiliations:** 1 Department of Neuroscience, Thomas Jefferson University, Philadelphia, PA USA; 2 Vickie & Jack Farber Institute for Neuroscience, Thomas Jefferson University, Philadelphia, PA USA; 3 Department of Psychiatry and Human Behavior, Sidney Kimmel Medical College, Thomas Jefferson University, Philadelphia, PA USA; 4 Department of Neurology of the Second Affiliated Hospital, Interdisciplinary Institute of Neuroscience and Technology, Zhejiang University School of Medicine, Hangzhou, China; 5 Department of Computer Science, Hertford College, University of Oxford, Oxford, UK; 6 Cold Spring Harbor Laboratory Cancer Center, Cold Spring Harbor, NY USA; 7 Department of Neuroscience, University of Rochester Medical Center, Rochester, NY USA; 8 Department of Psychiatry and the Behavioral Sciences, University of Southern California, Los Angeles, CA USA

**Keywords:** Synaptic development, Cellular neuroscience, Genetics of the nervous system

## Abstract

Since the introduction of second-generation antipsychotics, antipsychotics have been increasingly prescribed for children and adolescents, raising concerns about their long-term impact on neurodevelopment. Antipsychotics block dopaminergic and serotonergic receptors, potentially disrupting the maturation of neurocognitive processes, which is a public health concern. Previous studies have reported that adolescent antipsychotic treatment can cause persistent neurocognitive dysfunction in rodents, yet the neurobiological underpinnings remain unknown. To address this, we administered risperidone, a commonly used antipsychotic, to C57BL/6 mice during adolescence (3 to 6 weeks of age) and examined behavioral and neurobiological outcomes nine weeks post-treatment. Risperidone-treated mice exhibited subtle deficits in behavioral correlates of anxiety-like behavior. In vivo, two-photon calcium imaging of cortical neurons revealed a remarkable increase in the amplitude of calcium events with subtle sex-specific changes in the frequency, consistent with increased neuronal excitability. Single-nucleus RNA-sequencing (snRNA-seq) analyses showed widespread reductions in transcripts for voltage-sensitive and inwardly rectifying potassium channels in both pyramidal neurons and interneurons. Additionally, both cell types exhibited reduced *Grin2a* and *Grin2b*, as well as scaffolding proteins, indicative of weakened synaptic connectivity between excitatory and inhibitory neurons. Interestingly, we observed sex-dependent differences in the directionality of correlation between certain gene co-expression modules and risperidone treatment. Our results suggest that adolescent risperidone treatment induces lasting transcriptomic and functional changes associated with altered excitatory-inhibitory neuronal interactions that may underline cognitive and behavioral dysregulations.

**Figure:**
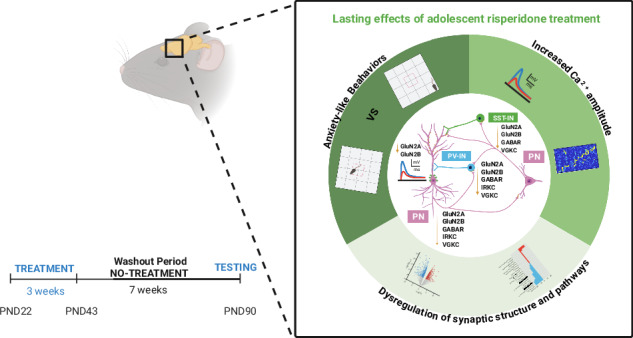

## Introduction

Antipsychotic use in children and adolescents has increased dramatically over the last decade in the U.S [1, 2], now affecting almost 2% of persons under the age of 18. Antipsychotics are prescribed for a variety of non-psychotic illnesses and off-label uses [2–8], raising concerns about long-term effects on the developing brain. Adolescence is a critical period for the maturation of higher-order cognitive functions, e.g., working memory and executive function [9, 10], driven by maturation of excitatory and inhibitory (E/I) balance and synaptic connectivity [11–13]. Disruption of these processes through adolescent antipsychotic exposure has the potential to lead to enduring neurodevelopmental effects.

During adolescence, the activity of interneurons (INs) becomes more robust, suppressing the spontaneous activity of pyramidal neurons (PNs) and enhancing their stimulus-driven neuronal activation. This refines the interactions between PNs and INs, which are essential for the coordinated development of distinct circuits and specific brain networks that are linked to higher-order cognitive functions.

Currently prescribed antipsychotics are often classified into three generations based on their receptor binding profiles [14]. First-generation antipsychotics (FGAs) achieve their therapeutic effects primarily by blocking dopamine D_2_ receptors (D_2_R). Second-generation antipsychotics (SGAs) also block D_2_Rs but extend their mechanism of action to other dopaminergic (DA) and serotonergic (5-HT) receptor subtypes, notably 5-HT_2_A. The most recently developed class, often called third-generation antipsychotics (TGAs), acts on DA and 5-HT receptors similarly to SGAs; however, they incorporate partial agonism rather than total blockade. Additionally, while profiles vary among individual drugs, SGAs generally exhibit higher affinities for D_3_R and D_4_R than FGAs. Similarly, certain SGAs and TGAs show higher affinity for 5-HT_6_ and 5-HT_7_ receptors compared to FGAs. Because these DA and 5-HT receptor subtypes are expressed across various brain regions following specific developmental trajectories [15, 16], antipsychotics of each generation may have differential impacts depending on the brain region and the stage of the lifespan.

Studies of the cognitive effects of antipsychotics in children and adolescents are lacking. A recent meta-analysis of the cognitive effects of antipsychotics in the treatment of adults with schizophrenia concluded that antipsychotics as a class are not procognitive agents [17] as have been reported in some prior studies. Indirect evidence of the cognitive impacts of antipsychotics in adults comes from studies of antipsychotic dose reduction. A meta-analysis of antipsychotic dose reduction in adults, comprised mainly of studies of FGAs, reported improved neurocognitive function with decreasing antipsychotic doses [18] with limited data for later classes of antipsychotics. A single- blinded dose reduction randomized controlled trial of adults with schizophrenia reported that reductions in risperidone or olanzapine doses were associated with improvements in processing speed and working memory [19].

Moreover, the dopaminergic and serotonergic systems mature in the association cortex during adolescence [20–22]. The expression of dopaminergic and serotonergic receptors peaks during early adolescence, a period critical to the maturation of E/I balance and synaptic connectivity. Furthermore, perturbation of the serotonergic system during adolescence has been shown to reinstate certain critical periods [23, 24]. Thus, antipsychotic-mediated blockade of these receptors during adolescence could disrupt the establishment of E/I balance, synapses, and circuits.

Preclinical studies have shown that antipsychotic treatment during the adolescent period of development can produce behavioral phenotypes in juveniles distinct from those resulting from adult treatment [10, 25]. Bardgett et. al. reported persistence in adulthood of alterations in locomotor activity [10], some of which exhibit sex-specific differences [26]. Qiao et al. similarly found reduced avoidance responses and significantly lower PCP-induced hyperlocomotion in adult male rats previously exposed to risperidone [27, 28]. Adolescent antipsychotic exposure has also been linked to long-term cognitive impairments, including deficits in learning and memory as well as alterations in behavioral flexibility [29] and the conditioned avoidance response (CAR) [30, 31]. Moreover, olanzapine treatment during adolescence disrupts working memory, fear conditioning [32], and stress responsivity [33].

Despite these documented behavioral consequences, the neurobiological mechanisms underlying the persistent cognitive effects of adolescent antipsychotic treatment remain poorly understood. We hypothesized that alterations of DA and 5HT signaling induced by SGAs during adolescence may lead to enduring changes in the neurobiological substrates of cognitive function. Here, we investigated the long-term consequences of adolescent antipsychotic exposure by integrating changes in neurocognitive behaviors, neuronal activity, and gene expression in adult mice. Specifically, we examined cognitive behaviors that involve recognition and memory, which are critically developed in adolescence and neuronal activity and transcriptome in PNs vs. INs, which may underlie the development of cognitive function during adolescence. While these cognitive and neurobiological changes may involve multiple brain circuitry and regions, our study is focused on the prefrontal cortex.

## Materials and methods

### Animals

Both male and female C57BL/6J (WT) mice (Jackson Laboratory; 10–30 g, 3 weeks old at the start of experiments) were used in all studies. Animals were acclimated for at least 5 days prior to experimental procedures. All experiments were conducted during the light cycle. All animal procedures were performed in accordance with the National Institutes of Health Guide for the Care and Use of Laboratory Animals and were approved by the Institutional Animal Care and Use Committee (IACUC) of Thomas Jefferson University.

### Study design for long-term effects of adolescent risperidone exposure

**Fig. 1:**
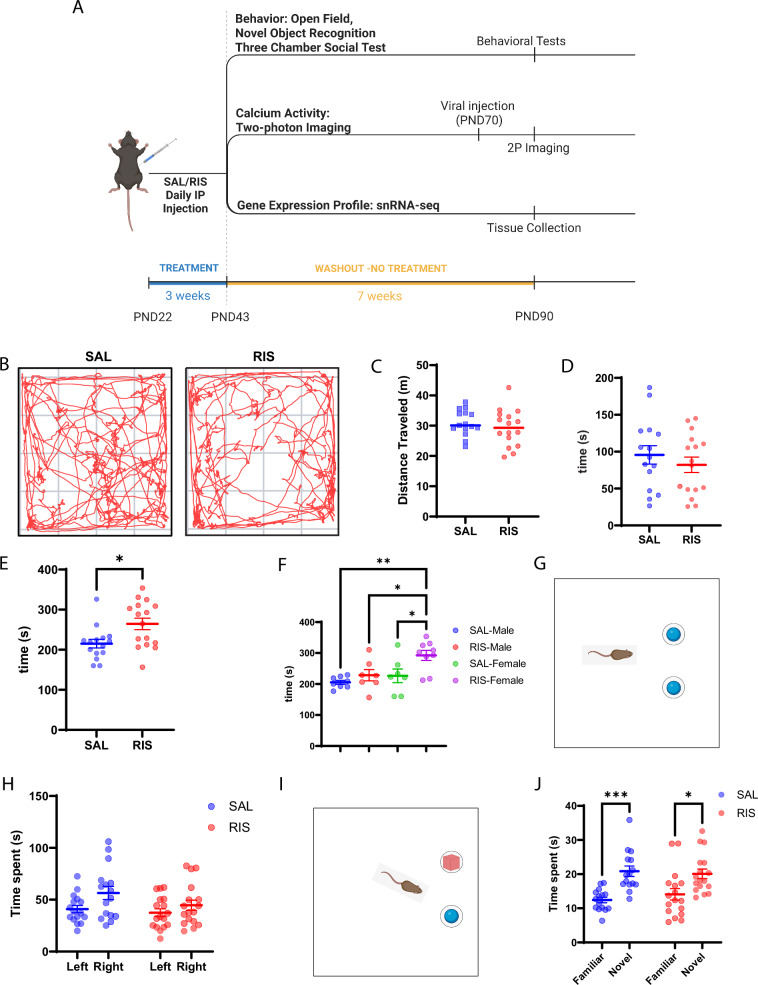
Adolescent risperidone treatment induced a long-term increase in anxiety-like behavior. **A** Schematic of the experimental timeline. **B** Representative trajectory of mouse movement within the OFT arena. **C** Total distance traveled (m) in the arena, **D** time spent in the center, **E** time spent in the corners, **F** time spent in the corners by sex. **G** Schematic of NOR test (P1), **H** Time spent exploring left vs. right objects. **I** Schematic during the recognition phase (P2), **J** time spent exploring familiar vs. novel object, (N: SAL M = 8, RIS M = 7, SAL F = 7, RIS F = 9).

To examine the long-term effects of adolescent risperidone exposure in adulthood, 3 week old adolescent WT mice received daily intraperitoneal (i.p.) injections of risperidone (4 mg/kg) [34] or saline from postnatal day (PND) 22 to 43, followed by a drug washout period from PND 43-90 (weeks 6-13) before the behavioral and neurobiological assessments (Fig. 1A). Washout periods of 3-10 weeks have previously been used in rodent studies [29–31]. A seven-week washout period was selected to evaluate the persistence of risperidone effects at a developmental stage in mice corresponding to human adulthood. Behavioral evaluations included the open field test (OFT), novel object recognition (NOR), and social interaction tests. These behavioral assessments were performed starting at PND 90. In parallel, cortical activity and molecular profiles were characterized using in vivo two-photon calcium imaging and snRNA-seq analyses (PND 90).

### Antipsychotic preparation

We focused on risperidone because it is one of the most extensively prescribed second-generation antipsychotics in child and adolescent populations. Additionally, it is well characterized pharmacologically, displaying potent D_2_ and 5-HT_2a_ antagonism. Risperidone (100 mg) was dissolved in 5 mL of DMSO to prepare a 20 mg/mL stock solution. The solution was gently heated and sonicated to ensure complete dissolution. A working solution was prepared daily by diluting 200 µL of the stock solution into 9.8 mL of sterile saline. Mice received 100 µL i.p. of the final solution per 10 g of body weight, corresponding to a dose of 4 mg/kg.

### Cranial window for chronic in vivo two-photon imaging

We performed surgeries two weeks prior to the end of the washout period (P70) to allow sufficient time for the AAV-mediated calcium biosensor to express. WT mice were prepared for surgery following established protocols [35]. A small (~1.5×1.5 mm) optical window was made by removing the thinned skull cap. 0.5 ﻿µL AAV virus AAV9-Syn-GCaMP6s-WPRE-SV40 (1.2 × 10^12^ copies/ml) was injected into the frontal cortical subregion M2. This infusion was performed from the pial surface using a micro-syringe pump as previously described [36]. See further details in the Supplementary Information.

### Two-photon Ca^2+^ imaging

Mice were recorded while awake, and calcium images were collected and analyzed according to published protocols [37]. Non-overlapping somas were selected manually as regions of interest (ROIs) and carefully inspected for overlap with other subcellular compartments. The mean fluorescence intensity of all ROIs was further analyzed. To reduce the influence of variability in background fluorescence and imaging conditions, relative changes in Ca^2+^ fluorescence (F) were calculated using the formula: ∆F/F_0_ = (F–F_0_)/F_0_ (with F_0_ = averaged fluorescence intensity over the entire trace) and used for all analyses of Ca^2+^ activity. The ∆F/F_0_ in the ROI region was considered as neuronal activity. Then, the events were detected based on published methods [38].

Because multiple cells were recorded from each mouse, the dataset had a hierarchical structure with individual cells nested within animals. To account for this non-independence, calcium event frequency and amplitude were analyzed using linear mixed-effects models in IBM SPSS Statistics (v29.0.1.0). Each model included treatment, sex, and the sex x treatment interaction as fixed effects, with subjects entered as a random intercept to model subject-level variability. Models were estimated using restricted maximum likelihood with a variance-components covariance structure and Satterthwaite approximation for denominator degrees of freedom. Statistical significance was defined as *p* < 0.05. See further details in the Supplementary Information.

### SnRNA-seq

For snRNA-seq experiments, mice were euthanized at PND 90, brains were harvested, flash frozen in liquid nitrogen, and cortical tissue samples were shipped to Cold Spring Harbor Laboratory (Cold Spring Harbor, NY) for snRNA-seq analysis. The right hemispheres of ten replicate cortexes (5 male, 5 female) from each condition (saline/risperidone) were minced with a razor blade on a metal block cooled by dry ice, and the pieces from each condition were combined into a single pool for nuclei prep. Nuclei were prepared from minced cortexes according to Martin et al. [39], stained with DAPI (0.1 µg/mL) and sorted on a SONY SH800 cell sorter with a 100 µm chip to remove non-nuclear debris. Sorted nuclei were pelleted at 600 x *g* and excess supernatant was removed and the remaining liquid (~50 µL) was used to resuspend nuclei. Single nuclei suspensions were counted and loaded into a Chromium X instrument, utilizing two technical replicate Chromium lanes per condition (4 lanes total) to achieve higher nuclei yields. Libraries were prepared using the GEM-X Universal Gene Expression v4 kit (1000691; 10X Genomics) and sequenced on a NextSeq2000 (Illumina) using the following read format: (28x10x10x90bp), to an average depth of ~16,000 reads per cell. Reads were aligned to the mouse reference genome GCRm38 GENCODE vM23 using Ensembl 98 annotations (i.e., prebuilt Cell Ranger reference mm10-2020-A, 10X Genomics, https://www.10xgenomics.com/support/software/cell-ranger/downloads/cr-ref-build-steps#mouse-ref-2020-a) using Cell Ranger v8.0.1 and default settings (which include assigning uniquely mapping intronic reads to their parent gene in the count matrix). The filtered count matrix produced by Cell Ranger’s cell-calling algorithm was used as input for downstream analysis. Male- and female-derived nuclei were later separated in silico using a gene expression signature.

### Statistical analysis

Data analysis was conducted using IBM SPSS Statistics (29.0.1.0), MATLAB R2024a (24.1.0.2628055), and GraphPad Prism 9 (9.5.1). The statistical tests were performed at a two-sided significance level of α = 0.05.

### Open field statistics

To assess treatment and sex effects on anxiety-like behavior, operationalized as time spent in the center and the corners of an open field arena, we conducted a two-way analysis of variance (ANOVA) using a general linear model framework. The dependent variable was total corner time, with treatment and sex included as fixed between-subject factors. The type III sums of squares were used to account for unbalanced group sizes.

In the event of significant main effects, post-hoc pairwise comparisons were conducted using estimated marginal means with Bonferroni correction to control for multiple comparisons. Effect sizes were reported as partial eta squared (ηp²) to quantify the magnitude of observed effects.

### Novel object recognition (NOR) statistics

We employed a repeated-measures analysis of variance (RM-ANOVA) within a general linear model framework to evaluate object exploration differences during the NOR task. Exploration time served as the dependent variable, with object (familiar vs. novel) included as a within-subject factor, and treatment and sex included as between-subject factors. Type III sums of squares were used to account for unbalanced group sizes.

Primary effects of interest included the main effect of object (i.e., preference for novel vs. familiar object), as well as interactions between object and treatment and/or sex. Significant main effects and interactions were further explored using estimated marginal means with Bonferroni correction.

Estimated marginal means and corresponding 95% confidence intervals were computed for each level of object, treatment, and sex, including higher-order interactions. Additionally, between-subject effects were assessed on the average exploration time across objects to evaluate overall differences in exploratory behavior independent of object preference.

### Two-photon Ca^2+^ imaging statistics

Calcium event frequency and amplitude were analyzed using linear mixed-effects models to account for the nested structure of the data (i.e., cells within animals). For each outcome measure, treatment and sex were included as fixed effects along with their interaction, and mouse identity was included as a random intercept.

Main effects and interactions were assessed using Type III tests of fixed effects. Post-hoc comparisons were performed using estimated marginal means with Bonferroni correction. Variance was examined to quantify within- and between-subject variability.

### SnRNA-seq statistics

For normalization and principal component analysis, nuclei were clustered using the Louvain algorithm at a resolution of 0.5. Gene Ontology (GO) enrichment analyses for upregulated and downregulated differentially expressed genes (DEGs) with an FDR threshold of <0.05 were performed for Biological Process (BP), Molecular Function (MF), and Cellular Component (CC) using the enrichGO function. Weighted gene co-expression networks were constructed using the soft power threshold determined via the Scale-free Topology Model (Figs. S10; S11), and the co-expression network modules were identified for glutamatergic neurons, PV-INs, and Sst-INs, which were visualized as dendograms. In order to compare the directionality of module-trait correlation in males to that of females, matching modules in different sexes were determined based on the overlapping genes in the top 100 hub genes as well as the Jaccard index between the two modules (Fig. S14).

## Results

### Adolescent risperidone treatment induced a long-term increase in anxiety-like behavior

The OFT was performed to evaluate general locomotor activity and anxiety-like behavior (Fig. 1B). When measured as the total distance traveled (m), locomotor activity did not differ between the risperidone and saline groups (Fig. 1C, dt (29) = 0.88, *p* = 0.39). Anxiety-like behavior was assessed by the total time mice spent in the center and in the corners of the field. While we found risperidone-treated mice didn’t spend significantly more time in the center, we observed that they spent significantly more time in the corners compared with saline-treated controls (Fig. 1D, E, F(1,27) = 7.386, *p* = 0.011, ηp²=.215), indicating increased anxiety-like behavior. Furthermore, when analyzed by sex, female mice showed an increase in the time spent in the corner relative to males (*F*(1,27) = 6.728, *p* = 0.015, ηp²=0.199), whereas the sex by treatment interaction was not significant *F*(1,27) = 1.718, *p* = 0.201, ηp²= 0.060 (Fig. 1F).

To test social motivation and preference, we employed the three-chamber social interaction paradigm, which consists of three sequential phases as described in the Supplementary Information. Across all three phases, the risperidone and saline groups exhibited comparable exploration and discrimination ratios (*t*(29) = 1.598, *p* = 0.1208, η² = 0.081). We found that sex by treatment interactions were not significant (Fig. S1).

In the NOR test, we recorded the duration of exploration of the novel vs. familiar objects. Both control and risperidone-treated animals displayed a novelty preference and therefore intact recognition memory. Neither treatment (*F*(1, 27–28) = 0.06–0.07, *p* = 0.80–0.82) nor sex (*F*(1, 27–28) = 0.33–0.40, *p* = 0.53–0.57) significantly altered novelty preference, and no treatment-by-sex interactions (*p* = 0.26) were observed (Fig. 1G–J).

### Adolescent risperidone treatment induced an increase in neuronal excitability in the adult mouse cortex

**Fig. 2:**
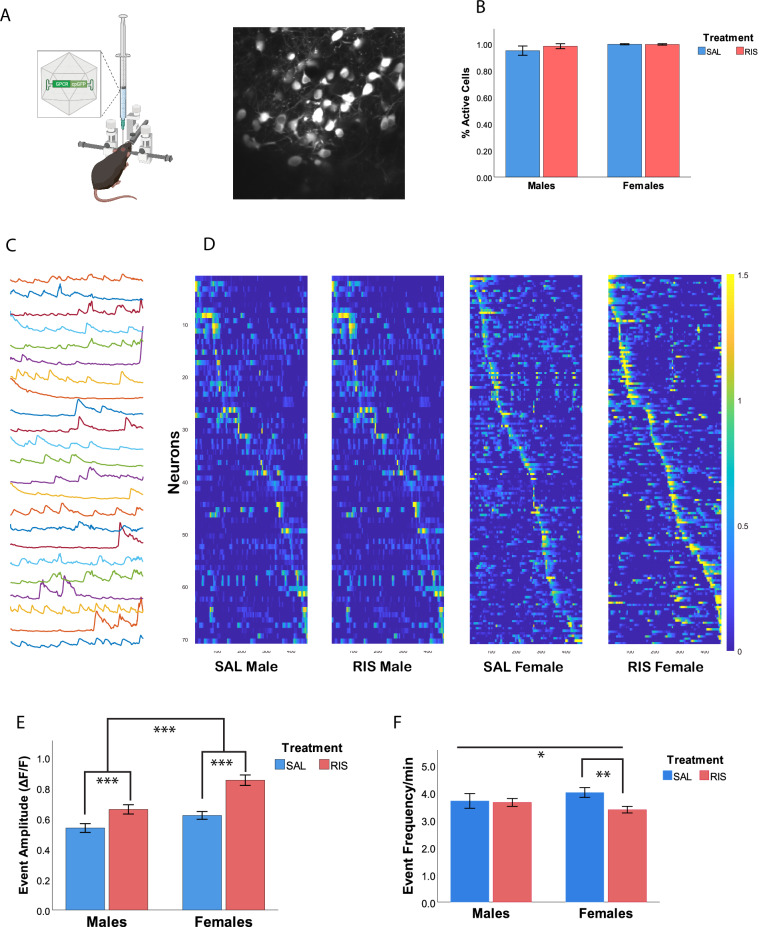
Adolescent risperidone RIS treatment alters neuronal calcium dynamics. **A** Schematic of a two-photon calcium imaging setup and a representative field of view showing GCaMP-labeled neurons. **B** Percentage of active cells by sex and treatment. **C** ΔF/F fluorescence traces from individual neurons during a 180-second spontaneous recording. **D** Heat maps of neuronal calcium activity (ΔF/F) across all recorded cells in SAL- and RIS-treated male and female mice. **E** Mean amplitude of calcium transients ΔF/F by sex and treatment. **F** Mean frequency of calcium transients by sex and treatment (N SAL M = 5, RIS M = 4, SAL F = 5, RIS F 5; N active cells: SAL M = 152, RIS M = 164, SAL F = 66, RIS F 158).

We conducted in vivo calcium imaging by injecting AAV-Syn-GCaMP6s into the secondary motor cortex (M2) and recording calcium transients in awake, head-fixed mice at PND90 (Fig. 2A). Fluorescence signals were quantified as relative changes in fluorescence (ΔF/F₀) and calcium transients were extracted as measures of neuronal activity (Figs. 2C, D and S2). The proportion of active cells among the identified was uniformly high across groups (~95–100%) and did not differ significantly by sex or treatment (Fig. 2B). Risperidone-treated mice exhibited profound alterations in calcium activity. Specifically, event amplitude was increased in risperidone-treated animals compared to saline controls (*p* < 0.001), with females showing higher amplitudes than males (*p* < 0.001) (Fig. 2E). Calcium event frequency was not significantly altered in risperidone-treated males (*p* = 0.375); however, there was a significant treatment-by-sex interaction (*F*(1, 541.96) = 4.07, *p* = 0.044). Post hoc analysis showed significantly reduced calcium event frequency in risperidone-treated females (*F*(1, 544.948) = 4.21, *p* = 0.041*) (Fig. 2F). Sex related differences for frequency in calcium event frequency reached nominal significance with *n*=4-5 animals per condition, and 152 active neurons in SAL-Male, 164 in RIS-Male, 66 in SAL-Females, and 158 in RIS-Females. In contrast, calcium amplitude showed robust main effects of sex and treatment. Amplitude has a larger effect than frequency, suggesting that alterations in synaptic strength and network excitability are more robust than firing rate dynamics.

### SnRNA-seq analysis shows transcriptional changes associated with synaptic function in risperidone-treated mouse cortex

**Fig. 3:**
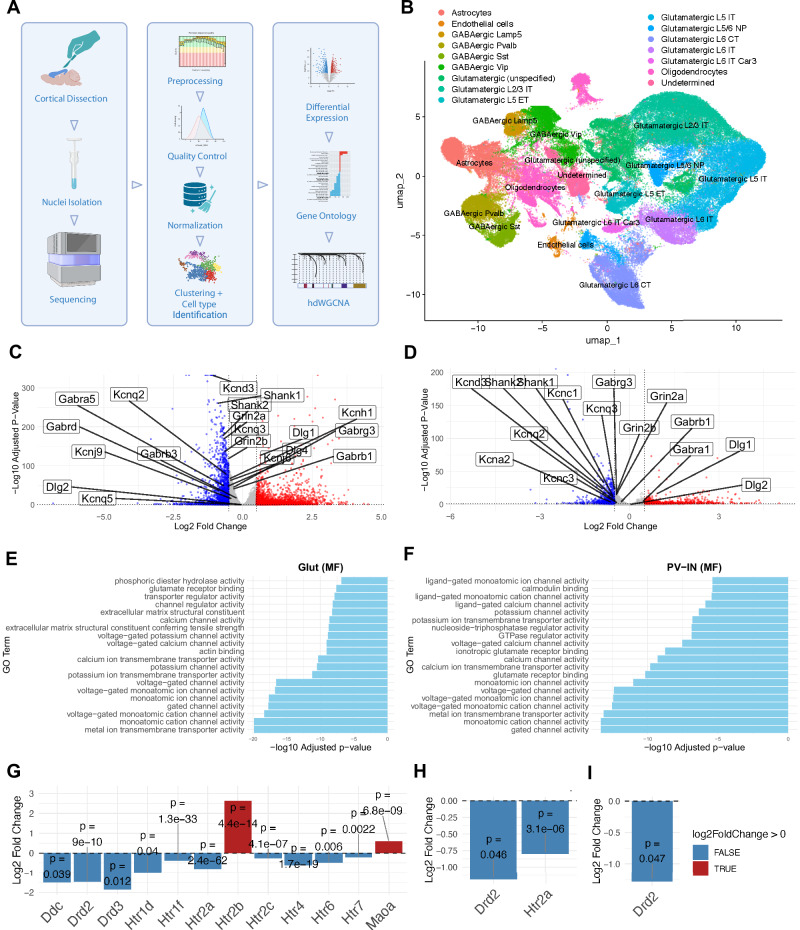
Adolescent risperidone treatment induces transcriptomic changes critical for neuronal excitability and synaptic connectivity in glutamatergic neurons and INs. **A** Schematics of snRNA-seq analysis pipeline. **B** UMAP visualization of snRNA-seq data with cell type annotation. IT, intratelencephalic; ET, extratelencephalic; NP, near-projecting; CT, corticothalamic. Volcano plots of DEGs in glutamatergic neurons (**C**), and PV-INs (**D**). Upregulated genes are in red and downregulated genes in blue. Horizontal dotted line represents -log10 adjusted *p*-value at 0.05, and vertical dotted line represents Log2FC at 0.5. Glut, glutamatergic neuron; PV-IN, parvalbumin expressing interneuron. **E**, **F** Top 20 GO enrichment terms in Molecular Function (MF) of downregulated DEGs in glutamatergic neurons (**E**), and PV-INs (**F**). Bar graphs of Log2FC of dopaminergic and serotonergic genes in risperidone-treated glutamatergic neurons (**G**), PV-INs (**H**), and Sst-INs (**I**). Only the statistically significant genes (FDR < 0.05) are graphed. FDR value for each gene is indicated.

A total of 27 clusters of nuclei were identified after employment of the Louvain algorithm (Figs. 3A and S3). Cell-type of each cluster was annotated manually based on the expression pattern of marker genes from literature [40] and visualized on the Uniform Manifold Approximation and Projection (UMAP) plot (Fig. 3B). We conducted pseudobulk differentially expressed gene (DEG) analysis between saline and risperidone groups, focusing on three cell types, glutamatergic neurons, PV- and Sst-INs (Fig. S4). These cell types were selected based on their critical roles in the interactions between PNs and INs. There were 1108 overlapping DEGs among the three cell types (Fig. S4E). Notably, these shared DEGs were enriched in Gene Ontology (GO) terms related to synaptic structure and function, and ion channel complex and activity (Figs. S4F–H). Similar GO term enrichment was observed in the downregulated DEGs (padj < 0.05, | log2FC | ≥0.5) in each cell type (Figs. 3C–F and S5). Specifically, voltage-gated potassium channels and inwardly rectifying channels were downregulated by risperidone (Figs. 3C, D and S4D) [41]. In addition, genes involved in glutamatergic and GABAergic synapses, particularly those that are integral to function and structure of the postsynaptic sites, were reduced by risperidone. Upregulated DEGs were enriched in terms associated with RNA metabolism and translation machinery in all three cell types (Figs. S5–7). For PV- and Sst-INs, few upregulated DEGs were also enriched in ion channel-associated terms (Figs. S6; S7). Notably, risperidone-treated mice showed downregulation of several dopaminergic and serotonergic receptor genes, particularly in glutamatergic neurons (Fig. 3G). Of these genes, *Drd2* was also downregulated in PV-INs and Sst-INs (Figs. 3H, I).

Since there were sex-dependent differences in neuronal activity, we performed DEG analysis in males and females separately for each cell type. In PV- and Sst-INs, higher numbers of genes were downregulated than upregulated, regardless of sex (Fig. S8). In all three cell types, genes related to voltage-gated potassium channels, glutamatergic and GABAergic synapses, as well as post-synaptic density were significantly altered (FDR < 0.05; Figs. S8; S9). However, no significant differences in genes related to synaptic connectivity were observed between males and females. We then asked if the genes that harbor the estrogen-responsive element (ERE) are differentially regulated by adolescent risperidone treatment between males and females. We found two genes that were altered in females and another two in males that were unaltered in the opposite sex (Fig. S18; Supplementary Table 3). The list of specific DEGs is further discussed in the Supplementary Results section, and complete lists of DEGs are found in the Supplementary Data Excel file 1.

### High-dimensional WGCNA identifies key gene modules associated with risperidone treatment

**Fig. 4:**
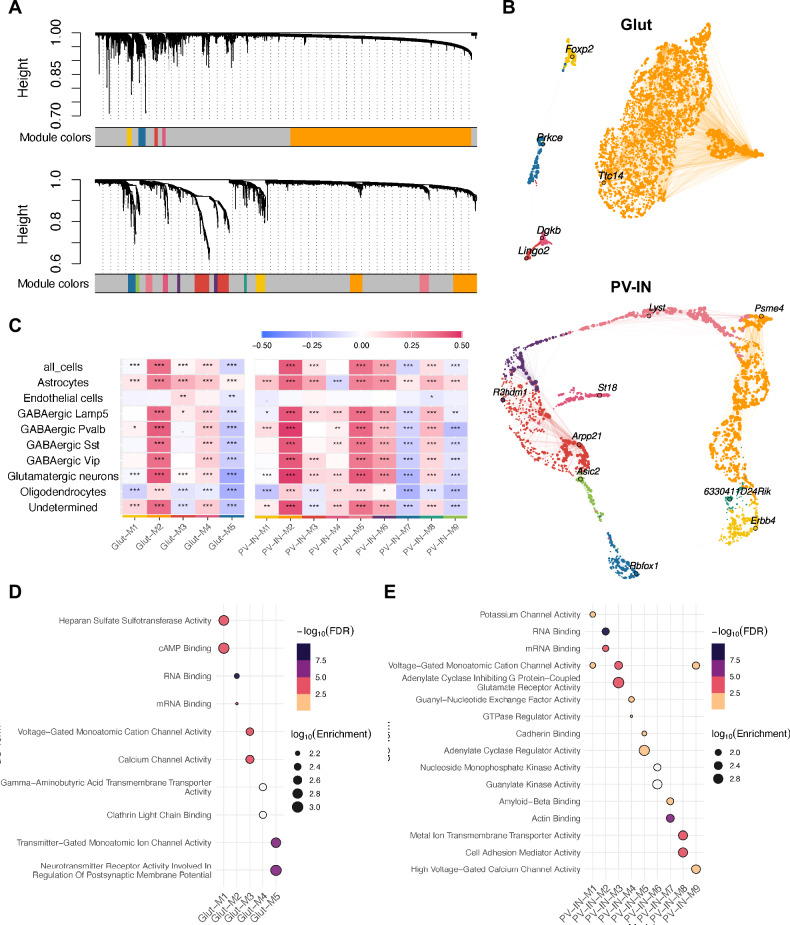
hdWGCNA analysis of glutamatergic neurons, PV-INs, and Sst-INs. **A** Dendogram of co-expression network for each cell type resulting in 5 modules in glutamatergic neurons (top) and 9 in PV-INs (bottom). **B** Gene co-expression network derived from Glut (glutamatergic neurons) and PV-INs, represented as a two-dimensional UMAP plot. Each node represents a gene, color-coded by module assignment, and its size is scaled by the gene’s kME. A top hub gene in each module is labeled. Edges represent the co-expression relationships between genes and module hub genes. For the purpose of visual clarity, edges were down-sampled to 10%. **C** Heatmap showing correlation between modules and treatment in different cell types, FDR < 0.1; *, FDR < 0.05; **, FDR < 0.01; ***, FDR < 0.001. The color bar shows the Pearson correlation coefficient. Dot plots representing top two GO enrichment results (Molecular Function) for each co-expression module for Glut (**D**) and PV-INs (**E**). Closed circle represents FDR < 0.05, and open circle represents FDR ≥ 0.05.

To identify key co-expression modules of adolescent risperidone treatment, we performed high-dimensional weighted gene co-expression network analysis (hdWGCNA) [42] in each cell type. We identified 5 co-expression modules in glutamatergic neurons, 9 in PV-INs, and 11 in Sst-INs, as visualized in dendograms, UMAPs, and dot plots (Figs. 4A, B and S12A, B; S13). The eigengenes of each module were tested for their association with treatment (Figs. 4C and S12C). The co-expression modules that were negatively correlated with risperidone (Glut-M5, PV-IN-M7, PV-IN-M9 in Fig. 4C; Sst-IN-M7 in Fig. S12C) in different cell types were enriched in GO terms associated with ion channel and neurotransmitter receptor activities (Figs. 4D, E and S12D). Modules that were positively correlated with risperidone (Glut-M2, Glut-M4, PV-IN-M1, PV-IN-M2, PV-IN-M4, PV-IN-M5, PV-IN-M6, PV-IN-M8 in Fig. 4C; Sst-IN-M2, Sst-IN-M3, Sst-IN-M4, Sst-IN-M5, Sst-IN-M10 in Fig. S12C) were enriched in terms associated with transmembrane transporter and channel activities, neurotransmitter receptor activities, as well as RNA processing and kinase/phosphatase activities (Figs. 4D, E and S12D), demonstrating that risperidone changes the behavior of co-expression network modules associated with various domains of synaptic activities in these cell types. Interestingly, we found sex-dependent differences in the module composition and the module-trait correlation. In all three cell types, some male modules showed differential correlation with risperidone compared to their matching female modules (Supplementary Table 1; Figs. S14–1^7^). Of note, the modules that showed sex-dependent differential correlation with risperidone were associated with postsynaptic density, channel, and neurotransmitter activities, suggesting that synaptic function regulation in these cell types may underlie the sex-dependent behavioral differences observed in risperidone-treated mice. Detailed sex-dependent module-trait correlation difference is further discussed in Supplementary Results and Supplementary Data Excel file 1.

## Discussion

Whereas previous rodent studies of adolescent antipsychotic treatment have reported long-term alterations in cognitive behaviors [9, 10, 27, 30–33], their neurobiological underpinnings remain largely unknown. Data presented here show robust alterations in neuronal activity and the transcriptomes of specific cell types, which are associated with changes in cognitive behaviors. Adolescent risperidone treatment has been shown to impair behavioral flexibility [29], conditional avoidance response [30, 31], working memory, fear conditioning, and stress responses [33]. In the present study, our findings regarding cognitive behaviors were subtle overall. While need-for-safety-like behaviors were significantly increased as measured by corner times during the OFT in risperidone-treated mice (Figs. 1E, F), social recognition or preference as assessed in the three-chamber test (Fig. S1), and NOR were unaltered (Fig. 1J).

Previous studies have shown that adolescent olanzapine treatment produces persistent increases in anxiety-like behaviors, in the absence of alterations in social behaviors [43, 44], findings that are closely aligned with those observed in the present study. In contrast, other studies have reported that adolescent olanzapine treatment causes enduring changes in cognitive domains: working memory and NOR [32, 33], effects that were not observed in our study. The reason for this discrepancy is presently unknown, but it may reflect key methodologic differences between studies, including species (rats vs. mice), drug choice (olanzapine vs. risperidone), and the duration of washout periods.

Compared to these changes in behaviors, we found robust effects of adolescent risperidone treatment on neuronal activity and transcriptomes. In vivo calcium imaging results demonstrate striking changes in cortical neuronal activity, specifically an increase in the amplitude of calcium activity events (Fig. 2E). Because the amplitude of the calcium activity reflects accumulated intracellular calcium from closely spaced action potentials and plateau depolarization [45], a robust increase in the amplitude in the risperidone treated mice suggests enhanced neuronal excitability [46]. While increased calcium events are often accompanied by decreased frequency [47, 48], which can mitigate the impact of increased amplitude, we found no change in the frequency of the events in male risperidone-treated mice (Fig. 2F). In addition, there was no change in the number of neurons with active calcium events during recording. Thus, the increased excitability in neurons of risperidone treated mice is likely to persist beyond the time point of this study.

We focused our snRNA-seq analysis on three neuronal cell types, glutamatergic, PV- and Sst-INs. These cell types were selected for their critical roles in the development of the E/I balance. DEGs resulting from adolescent risperidone treatment are enriched in specific pathways that affect interactions between excitatory and inhibitory neurons. While the DEGs generally differed across these cell types, we found a striking parallel in the most enriched pathways of DEGs of these cell types (Figs. 3 and S5–7). Specifically, the voltage-sensitive potassium channels were downregulated across these cell types, and inwardly rectifying potassium channels were downregulated in glutamatergic neurons and PV-INs (Supplementary Table 2). These extensive changes, when translated into downstream effects, could destabilize the resting membrane potential, desynchronize oscillatory coherence, and enhance neuronal excitability.

Furthermore, all three cell types showed downregulation of GluN2 subunits and scaffolding proteins, which could impact excitatory inputs into PNs and INs. Coupled with decreased GABA receptor expression in these cell types, the observed changes may lead to reduced excitatory input onto INs and/or weakened inhibitory control over PNs.

Co-expression module-based analyses identified the pathways functionally related within each experimental group (i.e., cell type or sex). Modules that were negatively correlated with risperidone, Glut-M5 in glutamatergic neurons, PV-IN-M7 & PV-IN-M9 in PV-INs and Sst-IN-M7in Sst-INs, were enriched for ion channels and neurotransmitter receptors (Figs. 4 and S12). This further supports the notion that adolescent risperidone treatment perturbs these pathways across all three cell types.

In addition, there appear to be pathways that are affected in a cell-type-specific manner. In glutamatergic neurons, Glut-M1 was also negatively correlated with risperidone, enriched in the heparan sulfate system and cAMP binding pathways. Heparan sulfate is crucial for the development of the extracellular matrix, neurons and synapses [49] while the cyclic AMP binding pathway is essential for neurotrophic processes, e.g., neurogenesis and synaptogenesis [50]. In interneurons, PV-IN-M2 and PV-IN-M5, positively correlated with risperidone, are enriched in RNA-binding and adhesion molecule pathways, suggesting robust activity of gene regulation and of cell-cell interactions.

Results from both DEGs and hdWGCNA modules highlighted dysregulation of biological pathways essential to synaptic structure and function. While DEG analysis demonstrated that key genes related to synaptic connectivity were dysregulated similarly in both sexes, sex-separated co-expression modules revealed that some gene groups were altered in divergent directions between males and females. It is important to note that gene expression profiles in neurons and their impact on synaptic and circuit activity are affected by changes in non-neuronal cells. Analysis of non-neuronal transcriptomic changes and their impact on synaptic changes is left for our future study.

Without an electrophysiological assessment of each cell type, our results do not directly inform the specific activity or balance of excitatory versus inhibitory neurons. However, our snRNA-seq data are consistent with a model in which glutamatergic neurons and interneurons are hyperexcitable, despite attenuated excitatory and inhibitory inputs to both. Interestingly, Badawi et al. [51] reported that acute risperidone treatment reduces the excitability of both neuronal types. Given that our findings represent enduring effects after chronic treatment, the opposite of these acute effects, this may reflect a compensatory reset of neuronal and synaptic activity via homeostatic plasticity.

As PNs constitute most cortical neurons, our snRNA-seq results likely relate to the observed increases in event amplitudes (Fig. 2E). Furthermore, synaptic interactions between PNs and INs within various circuits may be compromised. Such changes in specific brain circuits, e.g., the hippocampus–PFC axis, are expected to dysregulate neurocognitive functions of various domains, including working memory, affective experience, and executive functions that develop during adolescence.

To our knowledge, this is the first cell-type-specific analysis of enduring transcriptomic changes induced by adolescent antipsychotic treatment. Consequently, cross-referencing our data with comparable previous studies is difficult. Among the most relevant prior works is Younis et al. [52], which utilized bulk RNA sequencing after a 21-day clozapine treatment in adult mice followed by a 1-day washout. They reported 1037 altered transcripts, which overlapped with 478 DEGs in glutamatergic neurons, 136 in PV-INs, and 788 in Sst-INs (Supplementary Data Excel File 2).

Because controlled longitudinal studies of antipsychotics are not possible in humans, the study of antipsychotic effects in the human brain has relied on post-mortem analyses. For instance, Benjamin et al. [53] and Perzel Mandell et al. [54] compared the brain transcriptomes of schizophrenia patients who were on antipsychotics at the time of death with those who were not. Notably, these studies identified 910 transcripts in the striatum and 624 genes in the DLPFC, which overlapped with our identified DEGs in excitatory neurons (324 in the striatum; 108 in the DLPFC) (Supplementary Data Excel File 3).

Finally, it is important to note the extensive nature of the observed transcriptomic changes triggered by adolescent risperidone treatment, which affected thousands of genes. In contrast, antipsychotic treatment in adulthood altered only hundreds of transcripts [55, 56]. It is possible that these extensive transcriptomic changes are direct downstream effects of blockade of DA or 5HT receptors. These extensive changes may reflect a perturbation of the highly coordinated gene expression trajectory required for neuronal and synaptic maturation. Consequently, it is vital to investigate how the blockade of dopaminergic and serotonergic receptors during adolescence impacts the trajectory of brain development. If so, it will be important to investigate how receptor blockade of dopaminergic and serotonergic receptors during adolescence impacts the trajectory of brain development.

### Limitations

Our in vivo calcium imaging platform does not discern specific neuronal cell types. Our interpretation of calcium imaging data that the majority of PNs may be hyperexcitable is based on the notion that about 80 percent of neurons that are detected in our results are likely PNs. Cell-type-specific in vivo calcium imaging is needed to confirm our interpretation in future studies.

Our in vivo calcium imaging was derived solely from the M2 region in head-fixed awake mice, while behavioral phenotypes examined here involve various brain regions. Future studies should include region-specific calcium imaging during various behavioral tasks, such as fiber photometer analyses or mini scope-based paradigms.

There are many cognitive behavioral analyses that are not tested in this study. It will be important to monitor cognitive behaviors while measuring the activity in specific brain regions.

Treatment outcomes are determined by the dosage of agents and duration of treatment and washout period. To capture the full extent of possible consequences of robust treatment, we employed the dosage of risperidone that was close to the highest previously studied (4 mg/kg), as well as duration of treatment and wash-out periods that were comparable to the longest in prior studies.

## Supplementary information

Revised Supplementary Document

Supplementary Data Excel File 1

Supplementary Data Excel File 2

Supplementary Data Excel File 3

Supplementary Table 2

Supplementary Table 3

## Data Availability

The sequence and raw count matrices generated and analyzed for snRNA-seq experiments are deposited to the Gene Expression Omnibus, GSE316091 (https://www.ncbi.nlm.nih.gov/geo/query/acc.cgi?acc=GSE316091). All code used to generate results for snRNA-seq analysis is available at: https://github.com/Hahn-BorgmannWinter/Adolescent_antipsychotics.
