# Supplementary material for: Long-term effects of adolescent risperidone treatment on the mouse cortex
Source: Neuropsychopharmacology. 2026 Jul 8;51(10):1844–54. doi: 10.1038/s41386-026-02483-2 (PMC13486667; doi:10.1038/s41386-026-02483-2)
Supplement: Supplementary file 1 [file 41386_2026_2483_MOESM1_ESM.doc]

**Supplementary Information**

**Manuscript Title**: Long-term effects of adolescent risperidone treatment on mouse cortex

**Author List & Affiliations:** Abneil D. Alicea-Pauneto, B.S.*^1,2,^ Hyowon Choi, M.D., Ph.D.*^1,2,3^, Wenyu Zhang, Ph.D.^1,2^, Xinjian Li, Ph.D.^4^, Laurence. A. Busque, B.S.^1,2^, Mingxuan Li, Ph.D.^5^ Andrew Wu, B.S.^1,2^, Evan Zhang, B.S.^6^, Adam D. Marc, B.A.^1,2^ ,Jonathan Preall, Ph.D.^6^, Kuan Hong Wang, Ph.D.^7^,  Robert E. Featherstone, Ph.D.^8^, Steven J. Siegel, M.D., Ph.D.^8^, Chang-Gyu Hahn, M.D., Ph.D.^1,2,3^, and Karin E. Borgmann-Winter, M.D., M.S.^1,2,3^

**Supplementary Materials and Methods** 

***Animals***  
Animals were housed in groups of 4–5 per cage in a temperature-controlled room (22–24 °C) with relative humidity maintained at 40–60% under a 12 h light/dark cycle (lights on at 8:00 AM). Food and water were provided ad libitum.

***Behaviors***

*Open Field:* Exploratory behavior in a novel environment was evaluated using an open-field chamber measuring 43 x 43 cm. Each mouse was individually placed in the center of the arena, and its activity was monitored for 10 minutes. The session was recorded with a video camera, and the data were analyzed using the ANY-maze Video tracking system software (Version 7.34, Stoeling Co.).

The metrics analyzed during the test include the total distance traveled within the arena and the time spent in specific regions: the corner and the center. These regions were defined according to the grid overlay provided by the tracking software. The arena was divided into a 4 × 4 grid, with each grid square corresponding to approximately 10.75 × 10.75 cm. Corner zones were operationally defined as single grid-square regions located at each corner of the arena while the center was defined as 4 single grid-square located in the center of the arena. The testing environment was room temperature of 23°C.  

*Novel Object Recognition:* The test was performed to evaluate memory recognition and exploratory behavior in rodents, relying on their inherent curiosity toward novel stimuli. This test comprised two phases: during the training phase (1), mice explore two identical objects (two green cylinders, 4cm* 6cm) for 10 minutes, followed by an hour-long resting period. In between individuals, 70% ethanol was used to reduce olfactory cues from other mice. After an hour of rest in their home cages, mice are returned to the arena for the testing phase (2). Here, one familiar and one novel object (clear cube, 4cm*4cm*5cm) were presented, and exploration behavior was monitored and recorded for 10 minutes using video tracking software ANY-maze (Version 7.34, Stoeling Co.). Metrics such as novel object exploration time (sniffing) and discrimination indexes were calculated from the recorded data. The discrimination index was calculated as the ratio of the amount of time spent exploring the novel object over the total time spent exploring both objects.

*Three-Chamber Social Preference and Recognition Test*: The test, employed to evaluate social deficits in mice, utilized an opaque box measuring 52 × 26 × 23 cm, divided into three equally sized compartments: a central chamber and two side chambers (“Left” and “Right”). The test comprised three phases, each lasting 10 minutes. Phase 1 (Habituation) allowed mice to explore the chambers containing two empty wire cups on opposite chambers. In Phase 2 (Sociability test), one wire cup housed a stimulus mouse (the stranger mouse, matched for strain, age, and sex), while the other wire cup housed an inanimate object (clear block) in the other side chamber. Phase 3 (Preference for Social Novelty) replaced the inanimate object with a new mouse (Novel mouse, matched for strain, age, and sex). Exploration time was measured as the duration spent sniffing at the stimuli (object or mouse). Sniffing time was defined as the duration during which the animal’s head was oriented toward the wired cup and positioned within 3 cm of the cup. Animal behavior was recorded and analyzed using video tracking software ANY-maze (Version 7.34, Stoeling Co.). The discrimination ratio was calculated using the equation DR=(STL-STR)/(STL+STR), where STL and STR represent sniffing time for the left and right wire cups, respectively. The chamber was cleaned with 70% ethanol after each trial to prevent bias from olfactory cues.

***Cranial Window for chronic in-vivo two photon imaging***

The animal was maintained in a surgical plane of anesthesia throughout the procedure. A heating pad was used to maintain normal body temperatures. Surgical instruments are autoclaved before surgery, and the sterility of the instruments was maintained throughout the procedure with the hot bead sterilizer. The surgical instruments were sterilized using a hot bead sterilizer between animals. The mouse head was fixed on a stereotaxic apparatus. A scalp incision was made at the midline of the skull. The skull over the cortical region of interest was exposed, scrubbed, and cleaned. A high-speed dental drill (sterilized with hot bead sterilizer) was used to thin the skull, and the skull is periodically bathed in saline to dissipate drilling induced heat. 500 nL AAV virus, AAV-Syn-GCaMP6s-WPRE-SV40 (1.2×10^12 copies/ml), was infused into the subregion of the M2. This infusion was performed from the pial surface using a micro-syringe pump. The coordinates for infusion were determined relative to Bregma (AP 0.5, ML 0.5, and VD 0.5). The window then was sealed with a piece of glass coverslip (3 mm), a metal head bar, and dental cement. Mice received intraperitoneal injections of dexamethasone (5 mg/kg), Buprenorphine (0.1 mg/kg), and carprofen (5 mg/kg) to mitigate brain swelling and inflammation.

***Two-Photon Ca2+ Imaging***

Before each imaging session, mice were anesthetized with isoflurane using an induction chamber and gas regulator. They were then transferred to the microscope stage, where the implanted metal head bar was secured to the stage-mounted metal base to position the head beneath the objective. Isoflurane delivery was subsequently discontinued, and recordings were started 10 minutes after isoflurane was discontinued. Imaging was conducted using a two-photon laser-scanning microscope equipped with a laser system operating at 1000 nm, 80-MHz pulse repeat, and <100-fs pulse width (Newport laser system). The microscope was coupled to an upright Leica DM MLFS microscope and equipped with water-immersion objectives (×10, 0.3 numerical aperture (NA); ×20, 0.95 NA; ×40, 0.8 NA; ×60, 0.9 NA; all from Brand). The average power delivered to the brain was maintained at <70 mW.  After an image scan was completed (typically less than 30 minutes), the mouse was removed from the stage and returned to the home cage. The images were motion corrected and aligned. ‘Normcorre’ was then performed to correct the rigid and non-rigid motion of the images. For each imaging phase, images from all trials were concatenated into an image stack, subjected to bidirectional scan correction, and spatially down sampled by 2 × 2 pixels.  Registration across imaging phase: the mean intensity projections of each imaging phase were registered using ‘imregister’ in Matlab and the resulting shifts were applied to single frames. To select neuronal compartments, the ROI management tool of Image J was used (polygon selection for somas).

Calcium imaging videos were analyzed using custom-written scripts in MATLAB. The mean fluorescence intensity of each pixel during a recording session (around 2 min) was calculated as F0 and changes in pixel intensity at time *t*  were expressed as (Ft − F0)/F0 or ΔF/F0. To extract active dendritic signals, principal component and independent component analysis (PCA-ICA) was applied to the spatial-temporal data matrices of ΔF/F0 using CellSort and fastICA toolboxes (these toolboxes are freely downloadable from MATLAB Central). This analysis decomposes a spatiotemporal data matrix into independent components based on the skewness of data distribution. Each component has a characteristic spatial filter over the imaged area and a corresponding temporal signal during the imaging period. The spatial filter and the temporal signal of each component were graphically displayed and inspected by human observers who were blind to the experimental conditions of each video. If the spatial filter for a component overlapped with the dark shadows casted by blood vessels in the F0 image, this component was likely to be contributed by blood flow and was therefore rejected. In addition, because calcium signals show characteristic fast-rising and slow-decaying time course, the temporal skewness of calcium signals is expected to be positive and those components with skewness less than 1 were rejected. For each selected component, the location of the dendritic trunk was identified as the brightest spot (3 × 3 pixel) of the spatial filter. The corresponding temporal signal of the dendritic trunk was calculated from the ΔF/F0 video by subtracting the median value of the background area (outside the dendritic tree) from the average value of the dendritic trunk area.

***SnRNA-seq Analysis***

Nuclei from the cortex of 20 mice (10 risperidone and 10 saline treated;

5 males and 5 females in each group) that were sacrificed at PND90 were isolated (Figure 1). A total of 113,769 nuclei were sequenced, which were filtered for the number of unique genes and mitochondrial genes resulting in total of 109,585 nuclei (Figure S3F) for downstream analysis.

Analysis of snRNA-seq was performed and visualized within RStudio (v3.6.0) utilizing Seurat package (v5.2.0) [1] and clusterProfiler (v4.16.0) [2]. Quality control plots are included in Supplementary Figure 3.  Gene-level and cell-level quality control was completed by filtering nuclei based on thresholds of at least 250 genes detected, 500 UMIs, mitochondrial ratio below 20%, and gene complexity defined as log10(genes/UMI) > 0.8. Genes expressed in ≥10 nuclei were retained. This left a dataset that contained 48,700 nuclei for risperidone group and 60,885 nuclei for saline group. UMAP was used for non-linear dimensional reduction and visualization of the resulting 27 clusters. For cell type annotation, differentially expressed marker genes for each cluster were first identified using FindAllMarkers function. Cell-type of each cluster was manually annotated based on the known cell-type specific markers and neuronal meta-markers [3]. A cluster that could not be distinctly annotated was named “Undetermined.”

Pseudobulk differential expression analyses were executed using DESeq2 package (v1.48.2) [4] across the cell types of interest (GABAergic Pvalb⁺ and Sst⁺ GABAergic neurons and glutamatergic neurons) to elucidate the chronic transcriptional changes following risperidone treatment during adolescence. Sex-specific DE analyses were performed in each cell type after sub-setting the dataset based on the predicted sex.

High-dimensional Weighted Gene Co-expression Network Analysis (hdWGCNA) was performed using hdWGCNA R package (v0.4.07) [5]. Genes expressed at least 5% of the nuclei were included in the analysis. Module eigengenes (kME) and harmonized module eigengenes (hME) as well as module connectivity were computed. Genes in the co-expression network were visualized using UMAP with the top hub gene labeled in each module. Module-trait relationships were investigated to identify modules that are significantly associated with treatment conditions, which were visualized in heatmaps. GO enrichment analysis was conducted for each module and the top two enrichment terms visualized in dot plots. For the sex-dependent module-trait correlation analysis, co-expression network modules were first identified separately in male and female glutamatergic neurons, PV-INs, and Sst-INs, then the module connectivity and module eigengenes were computed. Module-trait relationships were determined separately in male and female neurons. Those modules that showed different directionality in their correlation with the RIS treatment were summarized in Supplementary Data Excel file1.

**Supplementary Results**

**DEG analysis between saline and risperidone groups**

Glutamatergic neurons showed 7,589 DEGs (P<0.01, FDR), with 3,864 downregulated and 3,725 upregulated genes (Figure 3C, S4A). In PV-INs, we found 1,616 DEGs (P<0.01, FDR), the majority of which were downregulated (Figure 3D, S4B). For Sst-INs, there were 878 DEGs (P<0.01, FDR), of which 664 were downregulated (Figure S4C, S4D).

In glutamatergic neurons, downregulated DEGs were enriched in GO terms associated with ion channels or synapse-related genes. Specifically, voltage gated potassium channels including *Kcna1*, *Kcna2*, *Kcna6, Kcnb1*, *Kcnb2*, *Kcnc1*, *Kcnc3*, *Kcnc4*, *Kcnd1*, *Kcnd3*, *Kcnh1*, *Kcnh3*, *Kcnh4*, *Kcnq2*, *Kcnq3*, *Kcnq4*, *Kcng1*, *Kcng2*, *Kcng3*, and inwardly rectifying potassium channels such as *Kcnj6*, *Kcnj12*, *Kcnj9*, *Kcnj4,* were identified. Additionally, genes that are involved in glutamatergic (*Grin1*, *Grin2a*, *Grin2b*, *Grin3a*, *Gria1*, *Gria3*, *Grid1*, *Grid2*, *Grik3*, *Grik4*, *Grik5*, *Grip1*, *Grip2*, *Grm1*, *Grm3*, *Grm5*, *Grm4*, *Grm8*, *Erbb4*, *Nrg1*, *Shank1*, *Shank2*, *Dlg3*, *Dlg4*, and *Dlg5*) and GABAergic (*Gabra5*, *Gabrb1*, *Gabrb3*, *Gabrg2*, *Gabrg3*, and *Gabrd*) synapses and signaling were included in the downregulated DEGs.

In PV-INs, similar RIS-dependent downregulation of voltage-gated potassium channels were observed: *Kcna2*, *Kcna6*, *Kcnb1*, *Kcnb2*, *Kcnc1*, *Kcnc2*, *Kcnc3*, *Kcnd3*, *Kcnq2*, K*cnq3*, *Kcnh1, Kcnh8*. Downregulated inwardly rectifying channels included *Kcnj6* and *Kcnj12.* Downregulated DEGs also included GABAergic and glutamatergic synapse related genes: *Gabrb1, Gabrd*, *Gabrg3, Grin2a, Grin2b,* *Grin2d*, *Grin3a*, *Gria1*, *Grid1*, *Grik1*, *Grik3*, *Grik4*, *Grik5*, *Grip1*, *Grip2*, *Grm4*, *Grm5*, *Shank1*, *Shank2, Dlg3*, and *Dlg5*.

In Sst-INs, RIS-dependent downregulated voltage-gated potassium channels included *Kcna6*, *Kcnb1*, *Kcnb2*, *Kcnc1*, *Kcnc2*, *Kcnc3*, *Kcnd3*, *Kcnq2*, *Kcnq3*. No inwardly rectifying potassium channel was significantly downregulated, though *Kcnj3* was upregulated by RIS in Sst-INs. Furthermore, similar to glutamatergic neurons and PV-INs, RIS downregulated several genes involved in glutamatergic and GABAergic synapse structure and function including *Grin1*, *Grin2a*, *Grin2b*, *Gria1*, *Grid1*, *Grik1*, *Grik3*, *Grik4*, *Grik5*, *Grip1*, *Grip2*, *Grm4*, *Grm5*, *Erbb4*, *Shank1*, *Shank2*,*Dlg5*, *Gabrb1*,*Gabrd*, and *Gabrg3*.

In sex-dependent DEG analysis, both male and female glutamatergic neurons showed RIS-dependent downregulation of potassium channel-related genes; *Kcna1, Kcna2, Kcna6, Kcnb1, Kcnb2, Kcnc1, Kcnc3, Kcnd2, Kcnd3, Kcng1, Kcng2, Kcng3, Kcnh1, Kcnh3, Kcnh4, Kcnk1, Kcnk2, Kcnk3, Kcnk9, Kcnk10, Kcnk12, Kcnk13, Kcnq4* in females, and *Kcna2*, *Kcna6*, *Kcnb1*, *Kcnb2*, *Kcnc1*, *Kcnc3*, *Kcnc4*, *Kcnd3*, *Kcng1*, *Kcng2*, *Kcng3*, *Kcnh1*, *Kcnh3*, *Kcnh5*, *Kcnh7*, *Kcnk1*, *Kcnk3*, *Kcnk9*, *Kcnk10*, *Kcnk12*, *Kcnq2*, *Kcnq3* in males (underlined genes are shared in both sexes). Several inwardly rectifying channels were also downregulated in both male and female glutamatergic neurons; *Kcnj6, Kcnj12, Kcnj9, Kcnj4*, except Kcnj3 in only males. Similarly, both male and female glutamatergic neurons showed downregulation of *Nrg1*, *Erbb4*, *Shank1*, *Shank2, Grin1, Grin2a, Grin2b, Grin3a, Gria1, Grid1, Grid2, Grik3, Grik4, Grik5, Grip1, Grip2, Grm1, Grm4, Grm5, Grm8, Dlg3, Dlg4,* and *Dlg5.* Female glutamatergic neurons additionally had downregulation of *Gria3*, *Grm7*, and *Dlg2*, while male glutamatergic neurons had downregulation of *Grik1* and *Grm3*. *I*n PV-INs and Sst-INs, similar downregulation of potassium channel genes and glutamatergic synapse related genes were observed in both males and females, though the number of genes were less than that of glutamatergic neurons. Complete list of DEGs are included in Supplementary Data Excel file 1.

**Sex-Dependent hdWGCNA Module-Trait Correlation**

There were several modules that showed differential correlation with RIS in male vs female. In glutamatergic neurons, modules between male and female neurons were matched based on their hub gene similarity. F_Glut-M8 (female glutamatergic neuron module 8) had positive correlation with RIS, while its matching module, M_Glut-M7 showed no correlation. F_Glut-M9 had negative correlation, while M_Glut-M8 had positive correlation. Additionally, M_Glut-M10 showed no correlation, while F_Glut-M1 showed negative correlation (Figure S15). In PV-INs, F_PV-IN-M3 was positively correlated, while M_PV-IN-M3 was negatively correlated (Figure S16). In Sst-INs, F_Sst-IN-M1 showed positive, but M_Sst-IN-M1 showed no correlation with RIS. F_Sst-IN-M3 had no correlation while M_Sst-IN-M5 had negative correlation. Similarly, F_Sst-IN-M6 had no correlation, while M_Sst-IN-M6 had negative correlation with RIS (Figure S17). List of GO enrichment terms and top 100 hub genes for these modules are included in Supplementary Data Excel file 1.

**Supplementary Figures**

**Supplementary Figure 1. Long-term behavioral effects of adolescent risperidone exposure on open field, three-chamber social interaction, and Novel Object Recognition performance.** (A) Body weight trajectories during 21 days of adolescent saline/risperidone treatment (RIS; 4 mg/kg) in male and female mice (N = 5).  Body weight increased significantly in all groups (mixed-effects model, REML; effect of day: F (2.112,8.446 = 59.02, p < 0.0001*).* No significant main effect of treatment/sex was detected (F(1.515,6.059) = 4.5, p = 0.0691) ) and analysis of day x group interaction was  non-significant as well (F (2.740,10) = 1.258, p = 0.3375), showing temporal trajectories were comparable between all groups. (B) Open field analysis of time spent in the center area, evaluated by treatment and sex. No significant main effects or interactions were observed. (C) Representative averaged heat maps illustrating exploratory trajectories for each treatment–sex group during the open field task as an additional visual representation of locomotor and exploratory behavior. (D) Schematic overview of the three-chamber social interaction paradigm used to assess the long-term effects of adolescent RIS exposure on social motivation and social recognition. (E) Phase 1: habituation/exploration phase. Mice were allowed to freely explore the arena containing two empty wire cups. (F) Phase 2: social preference phase. Animals were presented with a novel conspecific enclosed under one cup and a novel inanimate object placed under the opposite cup to evaluate social motivation. (G) Phase 3: social recognition phase. Mice were given the choice between a familiar conspecific previously encountered during Phase 2 and a novel conspecific to assess novelty-driven social recognition behavior. Across all phases, saline- and RIS-treated animals displayed comparable exploration patterns and discrimination indices, indicating that adolescent RIS exposure did not significantly alter adult social motivation or social recognition. (H) Quantification of Phase 3 exploration behavior revealed no significant treatment-, sex-, or interaction-dependent effects. (I) Paired exploration plots from NOR Phase 1 illustrating within-animal exploration behavior between the two empty chambers. (J) Paired exploration plots from NOR Phase 2 showing individual exploration preferences between the social stimulus and the object stimulus. (K) Treatment- and sex-stratified paired exploration analyses during NOR Phase 2, demonstrating tendencies and statistical comparable novel object preference behavior across all experimental groups. Neither treatment (F(1, 27–28) = 0.06–0.07, p = .80–.82) nor sex (F(1, 27–28) = 0.33–0.40, p = .53–.57) significantly altered novelty preference, and no treatment × sex interaction was observed (p = .26).

**Supplementary Figure 2. Quantification of labeled and active cells during two-photon calcium imaging.** Two photon calcium imaging was employed to study the effects of adolescent risperidone treatment on Ca transient transients in the M2 region of the cortex. (A) Number of active cells identified during calcium imaging, stratified by sex and treatment group (SAL vs. RIS). (B) Total number of labeled cells recorded during imaging sessions, stratified by sex and treatment group. Both measures revealed significant effects of treatment and sex on the number of cells detected.

**Supplementary Figure 3. SnRNA-seq data quality control.** (A) Number of UMI/transcript per cell. (B) Number of genes per cell. (C) Mitochondrial gene ratio per cell. (D) Correlation between genes detected (nFeature_RNA) and number of UMIs (nCount_RNA). (E-F) Number of nuclei in SAL and RIS group before the quality control (E), and after the quality control filtering (F). (G) Number of nuclei included in analysis per each annotated cell type.

**Supplementary Figure 4. Visualization of DEGs in glutamatergic neurons, PV-INs, and Sst-INs.** (A-C) Heatmap of DEGs (FDR < 0.05) between saline and risperidone group for glutamatergic neurons (A), PV-INs (B), and Sst-INs (C). (D) Volcano plot of DEGs (saline v. risperidone) in Sst-INs. Upregulated genes are in red and downregulated genes in blue. Horizontal dotted line represents -log10 adjusted p-value at 0.05, and vertical dotted line represents Log2FC at 0.5. (E) Venn Diagram of DEGs in three cell types. (F-H) Top10 GO terms of EnrichGO analyses of 1108 shared DEGs among three cell types for Biological Process (F), Molecular Function (G), and Cellular Component (H).

**Supplementary Figure 5. GO enrichment analysis of upregulated and downregulated DEGs in glutamatergic neurons.** (A-C) Top 20 GO terms of upregulated (red) and downregulated (blue) DEGs for Biological Process (BP) (A), Molecular Function (MF) (B), and Cellular Component (CC) (C) in glutamatergic neurons.

**Supplementary Figure 6. GO enrichment analysis of upregulated and downregulated DEGs in PV-INs.** (A-C) Top 20 GO terms of upregulated (red) and downregulated (blue) DEGs for Biological Process (BP) (A), Molecular Function (MF) (B), and Cellular Component (CC) (C) in PV-INs.

**Supplementary Figure 7. GO enrichment analysis of upregulated and downregulated DEGs in Sst-INs.** (A-C) Top 20 GO terms of upregulated (red) and downregulated (blue) DEGs for Biological Process (BP) (A), Molecular Function (MF) (B), and Cellular Component (CC) (C) in Sst-INs.

**Supplementary Figure 8. Heatmap of DEGs in male and female neurons.** (A, B) Heatmap of DEGs (FDR < 0.05) between saline and risperidone groups in female (A) and male (B) glutamatergic neurons. (C, D) Heat map of DEGs in female (C) and male (D) PV-INs. (E, F) Heatmap of DEGs in female (E) and male (F) Sst-INs.

**Supplementary Figure 9. Volcano plots of DEGs in male and female neurons.** (A, B) Volcano plot of DEGs (saline vs. risperidone) in glutamatergic neurons from female (A) and male (B) mice. (C, D) Volcano plot of DEGs in PV-INs from female (C) and male (D) mice. (E, F) Volcano plot of DEGs in Sst-INs from female (E) and male (F) mice. Upregulated genes are in red and downregulated genes in blue. Horizontal dotted line represents -log10 adjusted p-value at 0.05, and vertical dotted line represents Log2FC at 0.5.

**Supplementary Figure 10. Optimal soft power threshold selection for hdWGCNA.** (A-C) Scale-free topology model fit for different soft power values, and mean, median, and max connectivity for each soft power values for glutamatergic neurons (A), PV-INs (B), and Sst-INs (C). The lowest soft power threshold value that has a Scale Free Topology Model Fit greater than or equal to 0.8, and minimizing the Connectivity was selected for each cell type. Soft power threshold value of 22, 20, and 18 were used for glutamatergic neurons, PV-INs, and Sst-INs respectively.

**Supplementary Figure 11. Optimal soft power threshold selection for sex-specific hdWGCNA.** (A-F) Scale-free topology model fit for different soft power values, and mean, median, and max connectivity for each soft power values for female (A) and male (B) glutamatergic neurons, female (C) and male (D) PV-INs, and female (E) and male (F) Sst-INs. The lowest soft power threshold value that has a Scale Free Topology Model Fit greater than or equal to 0.8, and minimizing the Connectivity was selected for each cell type. Soft power threshold value of 10 (female) and 14 (male) for glutamatergic neurons, 10 (female) and 10 (male) PV-INs, and 10 (female) and 14 (male) Sst-INs were used.

**Supplementary Figure 12. hdWGCNA analysis of Sst-INs.** (A) Dendogram of co-expression network for Sst-INs resulting in 11 modules. (B) Gene co-expression network derived from Sst-INs, represented as a two-dimensional UMAP plot. Each node represents a gene, color-coded by module assignment, and its size is scaled by the gene’s kME. A top hub gene in each module is labeled. Edges represent the co-expression relationships between genes and module hub genes. For the purpose of visual clarity, edges were down-sampled to 10%. (C) Heatmap showing correlation between Sst-IN modules and treatment in different cell types. “.”, FDR <0.1; *, FDR < 0.05; **, FDR < 0.01; ***, FDR <0.001. The color bar shows the Pearson correlation coefficient. (D) Dot plots representing top two GO enrichment results (Molecular Function) for each co-expression module for Sst-INs. Closed circle represents FDR < 0.05, and open circle represents FDR ≥ 0.05.

**Supplementary Figure 13. Dot plot of co-expression module representation in different cell types.** (A-C) Module expression landscape across annotated cell types for the co-expression modules of glutamatergic neurons (A), PV-INs (B), and Sst-INs (C). Dot size represents the proportion of cells in each cell type with above-threshold eigengene score for that module. Color intensity represents the strength of module activity. Red means high eigengene expression, and blue means negative/low eigengene expression for that module.

**Supplementary Figure 14. Similarities between male and female modules.** (A-C) Heatmap of module similarity between male and female modules in glutamatergic neurons (A), PV-INs (B), Sst-INs (C). Color scale represents Jaccard index score. Number inside each box represents the number of overlapping genes in the top 100 hub genes of each module. *, Fisher’s exact p-value < 0.05.

**Supplementary Figure 15. Sex-specific hdWGCNA analysis of glutamatergic neurons.** (A) Heatmap showing correlation between glutamatergic neuron modules and treatment in different cell types for female (Left) and male (Right). “.”, FDR <0.1; *, FDR < 0.05; **, FDR < 0.01; ***, FDR <0.001. The color bar shows the Pearson correlation coefficient. (B, C) Dot plots representing top two GO enrichment results (Molecular Function) for each co-expression module for female (Left) and male (Right) glutamatergic neurons. Closed circle represents FDR < 0.05, and open circle represents FDR ≥ 0.05.

**Supplementary Figure 16. Sex-specific hdWGCNA analysis of PV-INs.** (A) Heatmap showing correlation between PV-IN modules and treatment in different cell types for female (Left) and male (Right). “.”, FDR <0.1; *, FDR < 0.05; **, FDR < 0.01; ***, FDR <0.001. The color bar shows the Pearson correlation coefficient. (B, C) Dot plots representing top two GO enrichment results (Molecular Function) for each co-expression module for female (Left) and male (Right) PV-INs. Closed circle represents FDR < 0.05, and open circle represents FDR ≥ 0.05.

**Supplementary Figure 17. Sex-specific hdWGCNA analysis of Sst-INs.** (A) Heatmap showing correlation between Sst-IN modules and treatment in different cell types for female (Left) and male (Right). “.”, FDR <0.1; *, FDR < 0.05; **, FDR < 0.01; ***, FDR <0.001. The color bar shows the Pearson correlation coefficient. (B, C) Dot plots representing top two GO enrichment results (Molecular Function) for each co-expression module for female (Left) and male (Right) Sst-INs. Closed circle represents FDR < 0.05, and open circle represents FDR ≥ 0.05.

**Supplementary Figure 18.  Transcripts of the genes that harbor Estrogen Response Elements (EREs) and were altered by adolescent RIS in PNs and INs.** DEGs of adolescent RIS treatment in specific cell types were surveyed for genes harboring EREs. PNs: Abcg2 and Ebag9 were increased only in females while Gad2, lgfbp4, Ovgp1 and Pgr were altered only in males. INs: No ERE-harboring genes were differentially altered between males and females.

**Supplementary Table 1. Female and their matching male modules that are differentially correlated with risperidone treatment.**

| Female module | Correlation with RIS | Matching male module | Correlation with RIS |
| --- | --- | --- | --- |
| Glutamatergic neurons | | | |
| F_Glut-M8 | + | M_Glut-M7 | Not correlated |
| F_Glut-M9 | - | M_Glut-M8 | + |
| F_Glut-M1 | - | M_Glut-M10 | Not correlated |
| PV-INs | | | |
| F_PV-IN-M3 | + | M_PV-IN-M3 | - |
| Sst-INs | | | |
| F_Sst-IN-M1 | + | M_Sst-IN-M1 | Not correlated |
| F_Sst-IN-M3 | Not correlated | M_Sst-IN-M5 | - |
| F_Sst-IN-M6 | Not correlated | M_Sst-IN-M6 | - |

**References**

1. Hao, Y., et al., *Dictionary learning for integrative, multimodal and scalable single-cell analysis.* Nat Biotechnol, 2024. **42**(2): p. 293–304.

2. Xu, S., et al., *Using clusterProfiler to characterize multiomics data.* Nat Protoc, 2024. **19**(11): p. 3292–3320.

3. Fischer, S. and J. Gillis, *How many markers are needed to robustly determine a cell's type?* iScience, 2021. **24**(11): p. 103292.

4. Love, M.I., W. Huber, and S. Anders, *Moderated estimation of fold change and dispersion for RNA-seq data with DESeq2.* Genome Biol, 2014. **15**(12): p. 550.

5. Morabito, S., et al., *hdWGCNA identifies co-expression networks in high-dimensional transcriptomics data.* Cell Rep Methods, 2023. **3**(6): p. 100498.
